# No relationship between inflammatory cytokines, heart rate variability, and morphology of the vagus nerves in patients with major depressive disorder

**DOI:** 10.1016/j.bbih.2025.101009

**Published:** 2025-05-08

**Authors:** Erik Scheller, Elise Böttcher, Lisa Sofie Schreiber, David Wozniak, Frank M. Schmidt, Johann Otto Pelz

**Affiliations:** a Department of Psychiatry and Psychotherapy, University of Leipzig Medical Center, Leipzig, Germany; b Department of Neurology, University of Leipzig Medical Center, Leipzig, Germany

**Keywords:** Depression, Microbiota gut brain axis, Vagus nerve, Ultrasound, Cytokines, Heart rate variability

## Abstract

Patients with major depressive disorder (MDD) often show of a low-grade inflammation. Inflammatory cytokines are assumed to be transmitted from the periphery to the brain, amongst others, via the vagus nerves (VN), which constitute a pivotal part of the microbiota-gut-brain axis. While functional aspects of the VNs (heart rate variability (HRV)) were extensively studied in patients with MDD, less is known about morphological alterations. Aim of this study was to examine the relationship between inflammatory cytokines, morphology, and function of the VNs in patients with MDD and healthy controls. Markers of inflammation (tumor necrosis factor alpha (TNF-alpha), interleukin 1 beta (IL-1 beta), and high-sensitive C-reactive protein (hsCRP)) were measured in 50 patients with MDD and 50 age- and sex-matched healthy controls. Inflammatory cytokines were correlated with sonographic characteristics of the VN (cross-sectional area and echogenicity) and with HRV at rest, during standing, and under slow paced breathing. Patients with MDD had significantly higher serum levels of IL-1 beta (0.17 ± 0.13 versus 0.09 ± 1.22 pg/ml, p < 0.001) and of TNF-alpha (0.72 ± 0.23 versus 0.62 ± 0.22 pg/ml, p = 0.013), while levels of hsCRP (1.91 ± 3.02 versus 1.60 ± 2.24 mg/l) were similar between groups. There was a significant correlation between body mass index (BMI) and hsCRP, as well as HRV parameters at rest in all participants. Controlling for the BMI, we found no correlation between inflammatory cytokines, HRV, and morphology of the VNs in patients with MDD. Therefore, further studies are warranted to address the assumed relationship between inflammation, morphology, and function of the VNs in patients with MDD.

## 1 Introduction

Major depressive disorder (MDD) is a highly prevalent and significant burden of disease and carries a high risk for recurrence and chronification (GBD 2019). Although the understanding of underlying mechanisms of MDD has progressed over the last decade, no single mechanism can explain every aspect of MDD (Malhi and Mann, 2018).

MDD is not only a mood disorder, but can also affect body health, especially cardiac, metabolic, and immune health (Tian et al., 2023). One of the most robust somatic findings in patients with MDD is a dysregulation of the autonomic nervous system with a relative sympathetic hyperactivity which is evident by an increased heart rate at rest (Kemp et al., 2010). Besides an overactivation of the sympathetic nervus system, there is also a reduced parasympathetic activity in patients with MDD resulting in a decreased heart rate variability under resting conditions and in an impaired response to autonomic stimulation tests (Kuang et al., 2019; Wu et al., 2023; Böttcher et al., 2024). This dysregulation of the autonomic nervous system is considered to be one reason for the increased risk for cardiovascular disease and even cardiac mortality in patients with MDD (Carney and Freedland, 2009; Jarczok et al., 2022; Zhao et al., 2023).

Interestingly, some characteristics of MDD like social withdrawal, loss of appetite, lethargy, or anhedonia resemble the so-called “sickness behavior” in severely ill, but in before mentally healthy, patients (Stieglitz et al., 2015; Turkheimer et al., 2023). A low-grade peripheral inflammation with elevated levels of multiple inflammatory serum cytokines was consistently found in patients with MDD (Yin et al., 2024). One of the best studied inflammatory cytokines in MDD is interleukin (IL) 1 beta which is frequently elevated in patients with MDD (Ellul et al., 2016). When injected directly into the rat hippocampus, recombinant human IL-1 beta was found to increase the serotonergic neurotransmission, the activity of the hypothalamic-pituitary-adrenocortical axis, and body temperature. Moreover, these rats also displayed typical characteristics of sickness behavior, such as immobility, piloerection, and a curled-up body posture (Linthorst et al., 1994).

However, it is not well understood how peripheral inflammatory cytokines reach the brain, which is shielded from the rest of the body by the blood-brain-barrier. One assumption is, that cytokines might enter the brain via leaky regions of the blood-brain-barrier such as the circumventricular organs, while another route of transmission might involve the vagus nerves (VN; Lichtblau et al., 2013; Miller and Raison, 2016). The VN with their afferent and efferent fibers form a bidirectional connection between the brain, the intestine, but also other organs like the heart (Wang et al., 2024). Cytokine receptors, mainly for IL-1 beta, were found in the peripheral vagal nerve sheath (Lichtblau et al., 2013), and cytokine-specific information was present in murine sensory vagal signals (Zanos et al., 2018). Recently, an impaired VN-mediated communication between the gut and the brain has gained more and more attention as a relevant factor in the pathophysiology of MDD (Tan et al., 2022; Yao et al., 2023). As outlined above, functional aspects of the VN like a reduced vagal (parasympathetic) tone were extensively studied in patients with MDD, however, less is known about structural changes of the VN. Recently, our group reported an enlarged cross-sectional area of the left VN in patients with MDD and in particular in the subgroup of patients with recurrent depressive disorder (Schreiber et al., 2023). Such a subtle enlargement of the cervical VN was also described in some rare acute inflammatory nerve disorders like in Guillain-Barré-Strohl (GBS) syndrome, where it was also associated with a reduced heart rate variability (Grimm et al., 2016). Noteworthy, patients with GBS syndrome had an increased risk of depression during the first two years after the diagnosis (Levison et al., 2023).

So far, research on the VN in patients with MDD focused on single aspects. Aim of this study was to explore the relationship between peripheral inflammatory cytokines, morphology and function of the VN in patients with MDD and healthy controls.

## 2 Methods

This study was performed according to the ethical standards laid down in the 1964 Declaration of Helsinki and its later amendment. It was approved by the local ethics committee of the Medical Faculty at the University of Leipzig (reference number 425/19-ek). All participants gave informed and written consent for participation in this medical research.

The original study comprised three predefined substudies. The first substudy focused on differences between patients with MDD and controls regarding the sonographic morphology of the VNs (Schreiber et al., 2023). The second substudy addressed the modulation of the autonomic nervous system in patients with MDD (Böttcher et al., 2024). Here, we report the results of cytokine measurements and their correlation with morphology and function of the VN. The protocol of this study ought to be presented at the “2nd Symposium of the Arbeitsgemeinschaft für Neuropsychopharmakologie und Pharmakopsychiatrie (AGNP) and Deutsche Gesellschaft für Biologische Psychiatrie (DGBP)” in 2020, which, unfortunately, was canceled due to SARS-CoV-2 pandemia (Schmidt et al., 2020).

Fifty patients with MDD and 50 age- and sex-matched healthy controls were recruited from June 2020 to September 2021 from the inpatient ward of the Department of Psychiatry and Psychotherapy, University of Leipzig Medical Center. All patients had to fulfill the clinical criteria of depression (F32.1-F32.2 and F33.1-F33.2) as defined by the International Statistical Classification of Diseases and Related Health Problems, 10th Revision). The diagnosis of MDD had to be confirmed by a senior psychiatrist. To evaluate the severity of depression at the time of participation, all participants completed the Beck Depression Inventory (BDI). At the time point of study participation, all patients in the MDD group were on antidepressants and were treated with psychotherapy.

Exclusion criteria were a medical history of polyneuropathy, epilepsy, neurodegenerative disorders, use of illegal substances, any addictive diseases, any psychiatric diagnoses in the control group, organic or psychotic psychiatric comorbidities, any relevant anxiety and/or obsessive compulsive disorders in the MDD group, a history of head injury, or acute somatic diagnoses during the time of examination.

### 2.1 Measurement of inflammatory serum markers

Based on previous studies and reviews by our research group (Lichtblau et al., 2013; Schmidt et al., 2016) but also by others (Harsanyi et al., 2022), we focused on established proinflammatory cytokines like IL-1 beta, IL-6, TNF-alpha, and high-sensitive C-reactive protein (Schmidt et al., 2020).

Blood samples of study participants were collected via venipuncture before the high-resolution ultrasound (HRUS) examination of the VNs and before measuring HRV. After collection, the serum was centrifuged at 5.400 rpm for 10 min, and the supernatant was stored at −80 °C until analyzes.

Serum levels of high-sensitive C-reactive protein (hsCRP) were determined by turbidimetric immunoassay “Cardiac C-Reactive Protein (Latex) High Sensitive” test and the cobas c® system (Roche Diagnostics Deutschland GmbH, Mannheim, Germany). The measuring range of the test was between 0.15 and 20.0 mg/l with an intra-assay coefficient of variation of <10 %.

Serum levels of interleukin 6 (IL-6) were analyzed by an electrochemiluminescence immunoassay (Elecsys® IL-6) and the cobas e® system (Roche Diagnostics Deutschland GmbH, Mannheim, Germany). The measuring range was between 1.5 and 5000 pg/ml, with a limit of quantification of 3.5 pg/ml (intermediate precision coefficient of variation of ≤20 %).

Enzyme-linked immunosorbent assays (ELISAs) were used to measure serum levels of interleukin 1 beta (IL-1 beta) and tumor necrosis factor alpha (TNF-alpha) according to the manufacturer's instructions (Human IL-1 beta/IL-1F2 Quantikine HS ELISA, assay range 0.1–8 pg/ml, and Human TNF-alpha Quantikine HS ELISA, assay range 0.2–10 pg/mL, respectively; both R&D Systems, Minneapolis, USA).

### 2.2 High-resolution ultrasound examination of the vagus nerves

Detailed information about the HRUS examination of the VNs were recently published (Pelz et al., 2018b; Schreiber et al., 2023). Briefly, the cross-sectional area (CSA) of the VN was measured at the level of the thyroid gland with an Aplio i800 (Canon Medical Systems, Neuss, Germany) with a 24 MHz linear transducer. In addition, the average echogenicity (grayscale mean, GSM) of the VNs with reference to the intraluminal common carotid artery (CCA) (GSM-VN/GSM-CCA) was determined.

### 2.3 Measurement of heart rate variability

Patients with atrial fibrillation, relevant cardiac arrhythmia, or intake of beta-blockers were excluded from HRV measurement. Measurement of HRV was previously described in detail (Böttcher et al., 2024). Briefly, after at least 5 min of resting in a calm environment, RR intervals were measured at normal breathing for 5 min under resting and in a supine position with the upper part of the body slightly elevated. Subsequently, participants stood up and measurement of HRV was repeated for 5 min while standing. For measurements of HRV under slow-paced-breathing (SPB), participants sat on a chair and breathed with a frequency of 6 breaths per minute (6 s of inhalation followed by 4 s of exhalation). The following HRV parameters were automatically computed: heart rate, the standard deviation of RR-intervals (SDNN), and the root mean square of successive differences (RMSSD).

### 2.4 Statistical analysis

Statistical analyses were performed by using IBM SPSS Statistics (IBM Corporation, Armonk, New York, USA; version 29.0). Shapiro-Wilk test was used to test for normal distribution. In case of a normal distribution, *t*-test was used for comparisons between groups and, while Mann-Whitney-U test was used in case of a non-normal distribution of data. Correlation analyzes were calculated with Pearson test (normal distribution) or Spearman signed-rank test (non-normal distribution). Chi-square test and Fisher's exact test were applied for group comparisons of nominally scaled data. The significance level was set at p < 0.05.

## 3 Results

Demographic data of patients with MDD and the control group were well-balanced in terms of sex and age. The body mass index was significantly higher in the MDD group (26.6 ± 5.7 kg/m^2^ versus 24.5 ± 3.6 kg/m^2^, p = 0.049; Table 1).

**Table 1:** Demographic data of patients with major depressive disorder (MDD) and healthy controls.

	MDD group	Control group	significance
Male (n (%))	21 (42 %)	21 (42 %)	1.00°
Female (n (%))	29 (58 %)	29 (58 %)	1.00°
Age (years, mean ± SD)	45 ± 17	46 ± 21	0.972#
BMI (kg/m^2^; mean ± SD)	26.6 ± 5.7	24.5 ± 3.6	0.049#
**Cardiovascular Risk Factors**			
Cardiac arrythmia (n (%))	5 (10 %)	1 (2 %)	0.092°
Diabetes mellitus (n (%))	3 (6 %)	1 (2 %)	0.307+
Smoking (n (%))	19 (38 %)	9 (18 %)	0.044°
Arterial Hypertension	11 (22 %)	10 (20 %)	0.806°
**Questionnaires**			
BDI score (median, range)	24.5 (6–46)	4 (0–20)	0.001#
Duration of actual depressive episode in weeks (mean ± SD)	25.39 ± 18.74^a^	0	
	4–80∗		

# Mann-Whitney-U-test; + Fisher's exact test, ° chi-square test; BMI body mass index, BDI Beck Depression Inventory, SD standard deviation.

a One extreme outlier of 400 weeks duration was excluded.

Measurement of IL-6 was futile, since in both groups most values were below the limit of quantification of 3.5 pg/ml. Patients with MDD had significantly higher serum levels of IL-1 beta (0.17 ± 0.13 pg/ml versus 0.09 ± 1.22 pg/ml, p < 0.001) and of TNF-alpha (0.72 ± 0.23 pg/ml versus 0.62 ± 0.22 pg/ml, p = 0.013) than healthy subjects, while levels of hsCRP were similar between groups (Table 2). Within the group of patients with MDD, patients with their first depressive episode had significantly lower levels of hsCRP than patients with recurrent depressive disorder (RDD; 0.7 ± 0.3 mg/l versus 2.6 ± 3.6 mg/l, p = 0.008). Levels of IL-1 beta and of TNF-alpha were similar between patients with their first or recurrent depressive episode (Table 3).

**Table 2:** Comparison of proinflammatory markers in the serum between patients with major depressive disorder (MDD) and healthy controls. ∗ Mann-Whitney-U test. hsCRP high-sensitive C-reactive protein, Il-1 beta interleukin 1 beta, TNF-alpha tumor necrosis factor alpha, SD standard deviation.

	Patients with MDD	Controls	Significance
hsCRP (mg/l, mean ± SD)	1.91 ± 3.02	1.60 ± 2.24	0.565∗
IL-1 beta (pg/ml, mean ± SD)	0.17 ± 0.13	0.09 ± 1.22	<0.001∗
TNF-alpha (pg/ml, mean ± SD)	0.72 ± 0.23	0.62 ± 0.22	0.013∗

**Table 3:** Demographic and proinflammatory serum markers of subgroups of patients with major depressive disorder. FD first time diagnosis, RDD recurrent depressive disorder ∗ Mann-Whitney-U test. ° chi-square test, hsCRP high-sensitive C-reactive protein, Il-1 beta interleukin 1 beta, TNF-alpha tumor necrosis factor alpha, BMI body mass index, BDI Beck Depression Inventory, SD standard deviation.

	Patients with FD (n = 18)	Patients with RDD (n = 32)	Significance
Male (n (%))	7 (38.9 %)	14 (43.8 %)	0.738°
Female (n (%))	11 (61.1 %)	18 (56.3 %)	
Age (years, mean ± SD)	39 ± 15	49 ± 17	0.044 ∗
BMI (kg/m2; mean ± SD)	24.2 ± 4.4	27.9 ± 5.9	0.037 ∗
BDI score (median, range)	27.5 (6–44)	23 (8–46)	0.968∗
hsCRP (mg/l, mean ± SD)	0.72 ± 0.34	2.58 ± 3.62	0.008∗
Il-1 beta (pg/ml, mean ± SD)	0.17 ± 0.12	0.17 ± 0.14	0.722∗
TNF-alpha (pg/ml, mean ± SD)	0.71 ± 0.21	0.73 ± 0.24	0.649∗

There was a strong correlation between the BMI and hsCRP as well as HRV parameters at rest in all participants, thus, we also controlled all correlation analyzes for the BMI. Within the group of patients with MDD there was no correlation between inflammatory cytokines and the sumscore of the BDI when controlling for the BMI (partial correlation, each p > 0.05). In both groups, TNF-alpha correlated with IL-1 beta (Table 4). We found no correlation between inflammatory cytokines and the sonographic morphology of the VN (Table 4). Neither in healthy controls nor in patients with MDD, we found a correlation between inflammatory parameters and HRV parameters at rest (Table 5). In healthy controls, hsCRP and TNF-alpha correlated significantly with SDNN and RMSSD during sympathetic challenge (standing; Table 5). Analyzing, the association between the sonographic morphology of the VN and HRV parameters, there was only a correlation between the echogenicity of the right VN and RMSSD and SDNN during sympathetic (standing) and parasympathetic (SPB) challenge in healthy subjects but not in patients with MDD (Table 6).

**Table 4:**
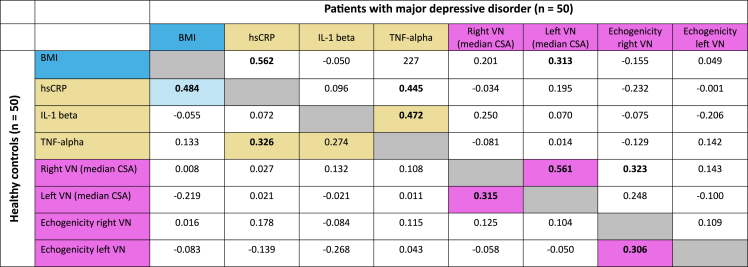
Correlation coefficients (rho) of inflammatory markers and vagus nerve (VN) high-resolution ultrasound parameters (cross-sectional area (CSA) and echogenicity) in 50 patients with major depressive disorder (upper part of the table, above the grey diagonal) and 50 healthy controls (lower part of the table, below the grey diagonal). Significant results (2-tailed) of the bivariate correlation (Spearman) are given in bold, while significant results (2-tailed) of the partial correlation with controlling for the body mass index (BMI) are given with a colored background. hsCRP high-sensitive C-reactive protein, Il-1 beta interleukin 1 beta, TNF-alpha tumor necrosis factor alpha, VN vagus nerve.

**Table 5:**
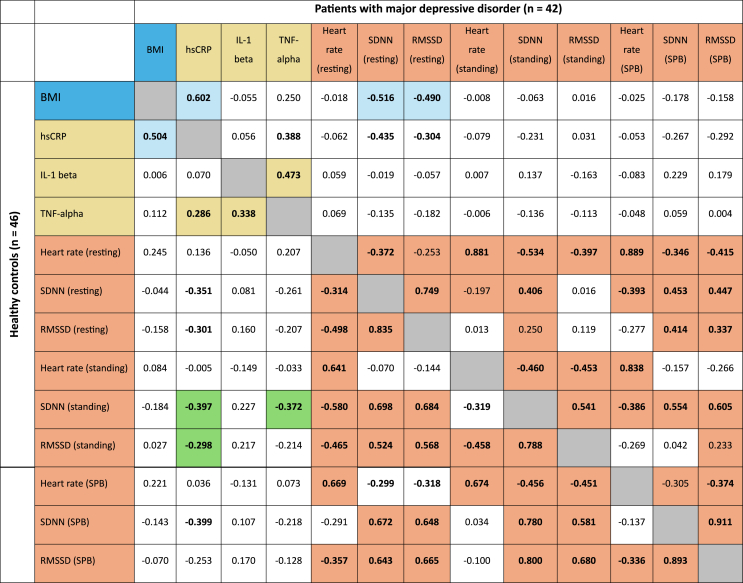
Correlation coefficients (rho) of inflammatory markers and heart rate variability parameters in 42 patients with major depressive disorder (upper part of the table, above the grey diagonal) and in healthy controls (n = 46, lower part of the table, below the grey diagonal). Participants with cardiac arrhythmia and/or intake of beta-blockers were excluded from heart rate variability analyzes. Significant results (2-tailed) of the bivariate correlation (Spearman) are given in bold, while significant results (2-tailed) of the partial correlation with controlling for the body mass index (BMI) are given with a colored background. hsCRP high-sensitive C-reactive protein, Il-1 beta interleukin 1 beta, TNF-alpha tumor necrosis factor alpha. SDNN standard deviation of RR-intervals, RMSSD root mean square of successive differences, SPB slow paced breathing, Sig significance.

**Table 6:**
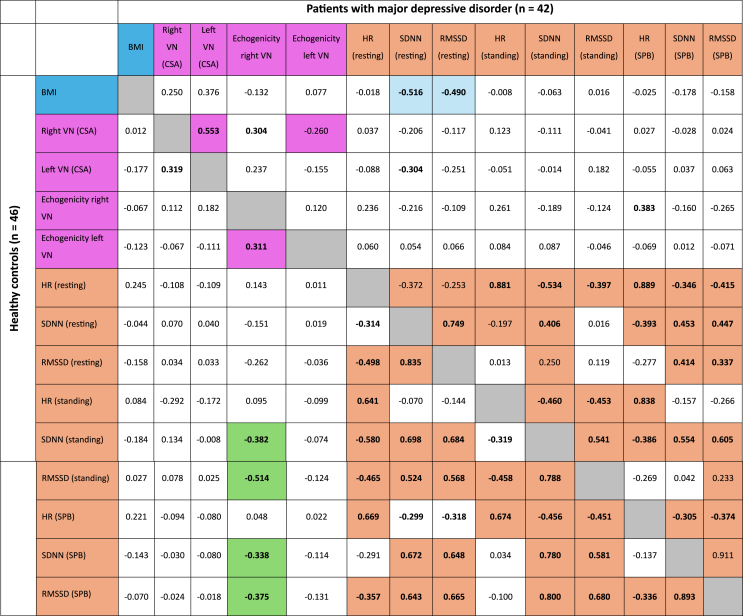
Correlation coefficients (rho) of vagus nerve (VN) high-resolution ultrasound parameters (cross-sectional area (CSA) and echogenicity) and heart rate variability parameters in 42 patients with major depressive disorder (upper part of the table, above the grey diagonal) and in healthy controls (n = 46, lower part of the table, below the grey diagonal). Participants with cardiac arrhythmia and/or intake of beta-blockers were excluded from heart rate variability analyzes. Significant results (2-tailed) of the bivariate correlation (Spearman) are given in bold, while significant results (2-tailed) of the partial correlation with controlling for the body mass index (BMI) are given with a colored background. SDNN standard deviation of RR-intervals, RMSSD root mean square of successive differences, SPB slow paced breathing, Sig significance.

In a general linear model with MDD/healthy controls as the dependent variable and cytokines, cross-sectional area of the VN, and HRV parameters as independent variables, there was only a trend for IL-1 beta (coefficient of regression 5.986, 95 % confidence interval −0.296 – 12.268, p = 0.062) to predict the state MDD/healthy control. Taking first time depressive episode/recurrent depressive disorder as the dependent variable, no parameter was significant.

## 4 Discussion

The main finding of this study was that patients with MDD had significantly higher serum levels of the proinflammatory cytokines IL-1 beta and TNF-alpha in comparison to healthy controls. Analyzing the associations between inflammatory cytokines, function, and morphology of the VNs, we found no clear relationship between these parameters.

Focusing on the microbiota-gut-brain interaction in MDD, translocation of bacterial components like lipopolysaccharide by a leaky gut would cause macrophages to release TNF-alpha and IL-1 beta (Tan et al., 2022). Both cytokines were shown to further loosen the tight-junction barrier of the gut, which might finally result in a sustained low-grade inflammation (Tan et al., 2022). Thus, our findings of elevated IL-1 beta and TNF-alpha serum levels were in line with these observations (Ellul et al., 2016). Interestingly, we found no correlation between the severity of MDD and the levels of proinflammatory cytokines. One explanation could be, that all patients were treated with antidepressants and psychotherapy, which might have mitigated the correlation. However, it is still unclear whether levels of proinflammatory cytokines correlate with the severity of MDD at all. Like in our study, Dahl and colleagues also reported no relationship between cytokines and severity of MDD (Dahl et al., 2014), whereas other researchers observed a positive correlation (Thomas et al., 2005), but also an even inverse correlation between inflammatory cytokines and severity of MDD (Schmidt et al., 2016).

Although the evidence of elevated levels of proinflammatory cytokines in the serum is robust, the mode of interaction with the brain has just recently been started to be elucidated. In mice, vagal afferents express receptors for TNF-alpha and IL-1 beta and, thus, can signal increased intestinal levels of these cytokines to the brainstem (Steinberg et al., 2016). The VNs not only sense a peripheral inflammation, but they can also elicit anti-inflammatory effects: via vagal efferents and the cholinergic anti-inflammatory pathway, and via vagal afferents, targeting the hypothalamic–pituitary–adrenal axis (Pavlov et al., 2003; Bonaz et al., 2017). Thus, stimulation of the VNs emerged as promising approaches to address inflammation in several disorders like MDD, cardiovascular disease, or inflammatory bowel disease (Bazoukis et al., 2023; D'Haens et al., 2023).

Recently, we observed an enlarged CSA of the left, but not of the right, VN in patients with MDD and especially in patients with recurrent depressive disorder (Schreiber et al., 2023). This enlargement was hypothesized to be due to a subtle edema of the VNs caused by the low-grade inflammation in MDD. However, here we found no relationship neither between the size of the (left) VN nor between the echogenicity of the VNs and serum levels of proinflammatory markers, which challenges this hypothesis. Structural changes of the VNs were observed in before in a variety of disorders: The VNs were found to be smaller in neurodegenerative disorders like Parkinson's Disease (Pelz et al., 2018a), and enlarged in autoimmune disorders like Guillain-Barré-Strohl syndrome (Grimm et al., 2016). Focusing on the microbiota-gut-brain interaction, an induced microbiota dysbiosis was reported to result in changes of the vagal structure, with a decrease of vagal fibers in the nucleus of solitary tract, that were accompanied by a loss of vagal function in rats (Kim et al., 2020). Since HRUS of the VN has been established over the recent years, further studies are warranted to explore a possible relationship between structure of the VNs and inflammation. In particular, since such a relationship might also be related to other proinflammatory cytokines like IL-2, IL-12, or interferon gamma.

The function of the VNs can be examined via measurement of HRV. HRV was robustly shown to be reduced in patients with MDD with an increased heart rate and decreased SDNN and RMSSD, the latter representing the vagal (parasympathetic) tone (Kemp et al., 2010; Wu et al., 2023). We also observed an increased heart rate at rest and during sympathetic and parasympathetic challenges and a decreased vagal tone in patients with MDD compared to healthy controls (Böttcher et al., 2024). Further exploring the relationship between morphology and function of the VNs in the current study, there was only an association between the echogenicity of the left VN and heart rate at rest and RMSSD in the small subgroup of patients with the first diagnosis of MDD. In a combined group of patients with Parkinson's disease and age-matched controls, RMSSD correlated with the CSA of the right but not of the left vagus nerve (Walter et al., 2018). Of note, even in healthy persons, findings of a relationship between structure and function of the VNs were inconsistent. Our group reported an inverse correlation between the *left* VN CSA and SDNN and RMSSD (Pelz et al., 2019), an inverse correlation between the *right* VN CSA and SDNN and RMSSD (Weise et al., 2022), and no correlation at all in the current study. These findings question the assumption of a relationship between function and structure of the VN, at least in healthy subjects, in general.

Finally, there was also no relationship between HRV parameters at rest and proinflammatory cytokines. In a meta-analysis, Williams and colleagues also reported no correlation between TNF-alpha and SDNN and RMSSD, as well as between IL-1 and SDNN (Williams et al., 2019). However, there was a significant inverse correlation between SDNN, RMSSD, and serum CRP. The authors state, that these differing findings might be due to the small sample sizes for IL-1 and TNF-alpha, while the correlation analyses for CRP and SDNN and for CRP and RMSSD, were based on over 16.000 datasets each (Williams et al., 2019).

This study has some limitations. All patients in our study were on antidepressant drugs. Since several effective antidepressants such as amitriptyline and mirtazapine have been shown to increase cytokine production (Lichtblau et al., 2013), while treatment with duloxetine reduced serum concentration of the proinflammatory cytokines IL-8, IL-12, and interferon gamma, but not of TNF-alpha or IL-1 beta (Gao et al., 2024), this might have affected the relationship between cytokine levels and severity of MDD as assessed via the BDI in our study. In case antidepressant treatment had lowered serum levels of inflammatory cytokines, this would also have affected the correlation analysis between levels of cytokine and both, HRV and sonographic morphology of the VNs. However, to control for the effect of antidepressants on cytokine levels, subgroups with intake of the respective antidepressant and an adequate sample size were needed, which would require a much larger study cohort. Another approach may be to examine antidepressant naïve patients, e.g. patients with a first episode. Secondly, the definition of depression as a disorder is based on symptoms forming a syndrome and causing functional impairment (Malhi and Mann, 2018). Thus, MDD is a clinical diagnosis with different underlying pathologies. Although the role of the microbiota-gut-brain axis in MDD gained more and more attention over the last years, its impact probably differs from patient to patient. This might finally have mitigated the assumed relationship between cytokines, vagal morphology and function. However, stratifying MDD according to the assumed underlying pathology is still a matter of debate and research. Thirdly, the effect sizes of HRV and ultrasound of the VN are typically small to medium with differences found on a group but less on an individual level. Thus, this study might have been underpowered for the detection of relationships between the examined parameters. Finally, this study was observational and cross-sectional, which limits causal inference. It cannot establish whether the observed cytokine levels or VN characteristics are causes or consequences of MDD. Longitudinal tracking of changes in VN morphology, cytokine levels, and HRV would provide insights into the temporal relationships and progression of these factors in MDD.

Concluding, although we were able to replicate important findings in patients with MDD like elevated proinflammatory cytokines and a decreased HRV, we did not find a relationship between markers of inflammation, HRV, and morphological alterations of the VNs in patients with MDD in comparison to healthy controls.

## Full data access and statements

Johann Otto Pelz as the Principal Author has full access to the data used in the analyses in the manuscript, and takes full responsibility for the data, the analyses and interpretation, and the conduct of the research.

## CRediT authorship contribution statement

**Erik Scheller:** Writing – review & editing, Investigation. **Elise Böttcher:** Writing – review & editing, Investigation. **Lisa Sofie Schreiber:** Investigation, Conceptualization. **David Wozniak:** Investigation. **Frank M. Schmidt:** Methodology, Conceptualization. **Johann Otto Pelz:** Writing – original draft, Methodology, Formal analysis, Conceptualization.

## Declarations

This manuscript complies with all instructions to authors.

All authorship requirements have been met and the manuscript was approved by all authors for publication.

This manuscript has not been published elsewhere and is not under consideration by another journal.

## Ethics approval

This cross-sectional study was performed according to the ethical standards laid down in the 1964 Declaration of Helsinki and its later amendment. The study was approved by the local ethics committee of the University Hospital Leipzig, Leipzig, Germany (reference number 425/19-ek). All study participants gave their written informed consent.

## Availability of data and materials

The dataset underlying this study is available from the corresponding author on reasonable request for any qualified investigator.

## Funding

No funding was received for this work.

## Declaration of competing interest

The authors declare that they have no known competing financial interests or personal relationships that could have appeared to influence the work reported in this paper.

## Data Availability

Data will be made available on request.
